# Gold Nanoparticle-Based Colorimetric and Electrochemical Methods for Dipeptidyl Peptidase-IV Activity Assay and Inhibitor Screening

**DOI:** 10.3390/ma9100857

**Published:** 2016-10-21

**Authors:** Ning Xia, Xin Wang, Xiaojin Wang, Binbin Zhou

**Affiliations:** Henan Province of Key Laboratory of New Optoelectronic Functional Materials, College of Chemistry and Chemical Engineering, Anyang Normal University, Anyang 455000, Henan, China; wangx933@nenu.edu.cn (X.W.); xjwanghxx@sohu.com (X.W.); xrc1202@gamil.com (B.Z.)

**Keywords:** dipeptidyl peptidase-IV, gold nanoparticles, colorimetric assay, electrochemical impedance spectroscopy, inhibitor screening

## Abstract

We presented the colorimetric and electrochemical methods for determination of the dipeptidyl peptidase-IV (DPP-IV) activity and screening of its inhibitor using gold nanoparticle (AuNP) as the probe. In the colorimetric assay, the substrate peptide with a sequence of Arg-Pro-Arg induced the aggregation and color change of AuNPs, whereas cleavage of the peptide by DPP-IV prevented the aggregation of AuNPs. Furthermore, the aggregation of AuNPs in the solution was easily initiated on a solid/liquid (electrode/electrolyte) surface, which induced a decrease in the electron-transfer resistance. However, once the peptide was clipped by DPP-IV, the assembly of AuNPs on electrode surface was prevented. Consequently, a higher electron-transfer resistance was observed. The colorimetric and electrochemical assays allowed for the determination of DPP-IV with the detection limits of 70 μU/mL and 0.55 μU/mL, respectively. Meanwhile, the proposed methods were used to determine DPP-IV inhibitor with satisfactory results. Both the colorimetric and electrochemical methods are simple, rapid and sufficiently sensitive for DPP-IV activity assay and inhibitor screening. The results also demonstrated that the AuNP-based colorimetric assay could be converted into an enhanced surface tethered electrochemical assay with improving sensitivity. The simple detection principle may be extended to the design of other peptidases biosensors with easy manipulation procedures.

## 1. Introduction

Diabetes is a group of metabolic disorder diseases. Type 2 diabetes, accounting for 90%–95% of all the cases, is characterized by high blood sugar, insulin resistance, and relative lack of insulin [1]. Recently, new therapies for managing this disease are focusing on modulation of the incretins glucose-dependent insulinotropic polypeptide (GIP) and glucagon-like peptide-1 (GLP-1), two peptidic hormones with glucose-dependent insulin secretion promotion and β-cell-proliferative effect [2,3]. However, these hormones show a rather short half-life to dipeptidyl peptidase-IV (DPP-IV, EC 3.4.14.5) [4]. Optimistically, DPP-IV inhibitors can be used to increase the lifetime of incretins by reducing DPP-IV activity, and DPP-IV has been regarded as the prime drug target to treat and prevent type 2 diabetes [2]. Although many of the inhibitors, such as sitagliptin, vildagliptin, and saxagliptin, exhibited impressive intrinsic potencies by lowering the level of serum glucose, these drugs also lead to some undesired side effects, such as angioedema, pancreatitis, and infective disorders [5,6]. Therefore, novel DPP-IV inhibitors were desired for the management of type 2 diabetes [7,8]. The currently used methods for monitoring DPP-IV activity and screening of its inhibitor include spectrophotometry, fluorescence, high performance liquid chromatography (HPLC) and mass spectrometry [9,10,11,12]. These methods are feasible, but are high-cost and time-consuming and require the use of labeled substrates and sophisticated instrumentations. Thus, the development of simple, sensitive and cost-efficient methods for probing of DPP-IV activity and screening of its inhibitor are still desired.

In recent years, gold nanoparticles (AuNPs) have been used as the excellent scaffolds for the design of chem/bio-sensors due to their distinct physical and chemical properties [13,14,15,16,17,18,19,20]. For example, because of their high extinction coefficients and the unique size-dependent optical properties, AuNP-based colorimetric assays have been reported for detection of many types of analytes, including monitoring the enzyme activity [21,22,23,24,25,26] and measuring the concentration levels of nucleic acids [27,28,29], proteins [30,31], metal ions [32,33,34] and other small molecules [35,36,37,38,39]. As to the assay of protease activity with AuNPs as the probes, two strategies could be commonly used. In the first method, peptide substrate containing two binding tags can crosslink AuNPs before the enzymatic reaction, thus inducing their aggregation and color change [24]. The second method is based on the non-crosslinking AuNPs aggregation in which the peptide substrate either before or after the cleavage reaction could cause the loss of surface charges of AuNPs and destroy the stability of the negatively charged AuNPs [25,26]. Since DPP-IV is a peptidase responsible for the cleavage of X-Pro-peptide, where X represents an amino residue, the previously reported AuNP-based colorimetric protease assays pave us a new way for the detection of DPP-IV by rationally designing the sequence of DPP-IV substrate.

**Scheme 1:**
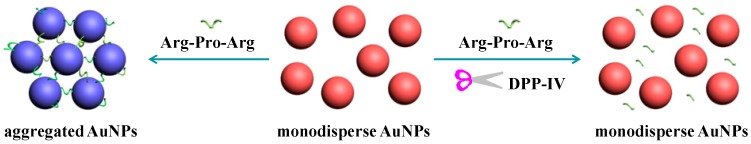
Schematic illustration of the colorimetric assay of dipeptidyl peptidase-IV (DPP-IV) activity using gold nanoparticles (AuNPs) as the probes.

**Scheme 2:**
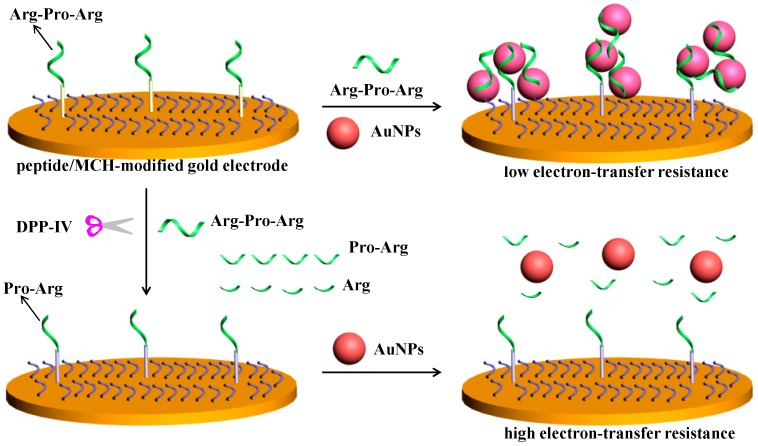
Schematic illustration of the electrochemical method for DPP-IV activity assay.

Herein, we found that the tripeptide (Arg-Pro-Arg) with two positively charged arginine residues could induce the aggregation and color change of AuNPs, whereas cleavage of the peptide into arginine and dipeptide (Pro-Arg) by DPP-IV prevented the aggregation of AuNPs (Scheme 1). This colorimetric methodology is simple because it does not require modification of analyte-binding molecules onto AuNPs. However, the colorimetric method showed poor sensitivity. Since gold electrode exhibits a superficial microenvironment similar to that of AuNPs [40], we suggested that modification of gold electrode with the peptide substrate allowed for the deposition of increasing amounts of AuNPs on the electrode surface through the tripeptide-induced assembly of AuNPs (Scheme 2), that is, the sensing electrode can capture AuNPs and free tripeptide in the solution through the peptide-AuNPs-tripeptide interaction. Then, surface-tethered peptide-AgNPs-tripeptide recruited more AuNPs as well as tripeptide, resulting in the formation of a network of AuNPs-tripeptide on the electrode surface. Thus, the aggregation of AuNPs in a solution would be facilely initiated on a solid/liquid (electrode/electrolyte) surface, yet cleavage of the peptide substrate either on electrode surface or in solution by DPP-IV prevented the assembly of AuNPs. Consequently, the liquid-phase colorimetric assay was converted into enhanced surface tethered electrochemical analysis for probing of DPP-IV activity.

## 2. Results and Discussion

### 2.1. Feasibility for Colorimetric Assay of DPP-IV

**Figure 1:**
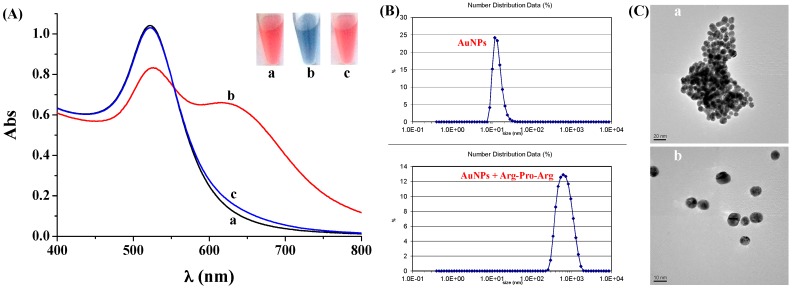
(**A**) UV–vis absorption spectra and photographic images of AuNPs in various systems (curve/tube a, AuNPs; curve/tube b, AuNPs + tripeptide; curve/tube c, AuNPs + tripeptide/DPP-IV). The final concentrations of AuNPs, tripeptide and DPP-IV were 3.6 nM, 5 μM and 250 mU/mL, respectively; (**B**) dynamic light scattering (DLS) of AuNPs in the absence and presence of tripeptide Arg-Pro-Arg; (**C**) TEM images of AuNPs in the presence of tripeptide (panel a) and tripeptide/DPP-IV (panel b).

To prove that the tripeptide Arg-Pro-Arg could be clipped by DPP-IV, the mixture of tripeptide/Arg-Pro-Arg was incubated for 1 h and then characterized by mass spectrometry in a positive-ion mode. As a result, two new dominant mass peaks at 175.1194 Da and 272.1741 Da were observed, corresponding to the positive-ion ESI–MS of Arg and Pro-Arg, respectively. Peptide-triggered color and absorbance change of AuNPs suspension in the absence and presence of DPP-IV was first investigated by naked eyes and UV-Vis spectrophotometer. As shown in Figure 1A, the red AuNPs solution (tube a) showed an absorption peak at 520 nm (curve a). Upon addition of the tripeptide Arg-Pro-Arg, the color of AuNPs suspension changed from red to blue (tube b). Meanwhile, the original absorbance of AuNPs suspension at 520 nm decreased while a new absorbance peak at ~630 nm appeared (curve b). Moreover, we found that the zeta potential of AuNPs changed from −43.9 to −22.1 with the addition of Arg-Pro-Arg, and their size increased from 13 nM to 615 nm by dynamic light scattering (DLS) (Figure 1B). The aggregation of AuNPs can be attributed to the electrostatic interaction between the negatively charged citrate-capped AuNPs and the positively charged arginine residues in the two terminals of the tripeptide [21]. However, once the tripeptide was pre-incubated with DPP-IV for a given time, the AuNPs suspension kept at red (tube c) and showed one absorption peak at 520 nm (curve c), indicating that the cleavage products of arginine and dipeptide Pro-Arg could not crosslink AuNPs and cause their aggregation. In fact, we also found that the mixture of arginine and dipeptide Pro-Arg at the concentration of 5 μM indeed did not induce the aggregation and color change of AuNPs suspension, further confirming the rationality of our design strategy. Furthermore, the tripeptide-triggered aggregation of AuNPs was verified by the TEM observation (Figure 1C). AuNPs aggregated significantly in the presence of peptide only (panel a), but they were monodisperse when the tripeptide was clipped by DPP-IV (panel b).

**Figure 2:**
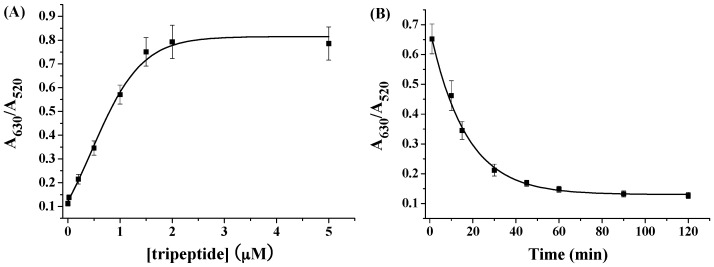
Dependence of the A_630_/A_520_ on the tripeptide concentration (**A**) and DPP-IV cleavage time; (**B**) The final concentration of AuNPs was 3.6 nM. The final concentrations of tripeptide and DPP-IV in pane B were 2 μM and 250 mU/mL, respectively.

To optimize the experimental conditions for DPP-IV assay, we first investigated the dependence of AuNPs aggregation on the tripeptide concentration. The A_630_/A_520_ ratio was used to evaluate the analytical performances, wherein A_630_ and A_520_ represented the absorption intensity of the AgNPs suspension at 630 nm and 520 nm, respectively. It can be observed that the A_630_/A_520_ increased linearly in the tripeptide concentration ranging from 0 to 1.5 μM (Figure 2A). Thus, the optimized concentration ratio of peptide to AuNPs was estimated to be 417:1. Cleavage time has a profound influence on the cleavage reaction. We also studied the influence of cleavage time on the absorbance change of AuNPs. The A_630_/A_520_ decreased with the increase of cleavage time (Figure 2B) and began to level off beyond 60 min. The result indicated that a sufficient reaction time is beneficial for a complete cleavage reaction. Thus, in the following detection assay, the peptide was allowed to be clipped by DPP-IV for 60 min.

### 2.2. Effect of DPP-IV Concentration and Activity on the Colorimetric Assay

**Figure 3:**
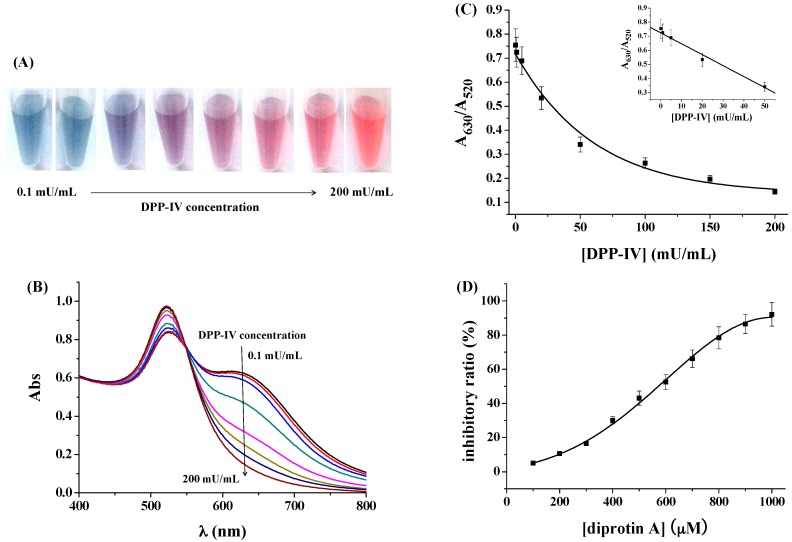
Photographic images (**A**) and UV–vis absorption spectra; (**B**) of AuNPs in the presence of peptide and various concentrations of DPP-IV (0.1, 1, 5, 20, 50, 100, 150 and 200 mU/mL); (**C**) Dependence of the A_630_/A_520_ on the DPP-IV concentration. The final concentrations of AuNPs and peptide were 3.6 nM and 2 μM, respectively; (**D**) Inhibitory ratio of different concentrations of diprotin A (100, 200, 300, 400, 500, 600, 700, 800, 900 and 1000 μM) on the DPP-IV (50 mU/mL) activity.

Under the optimal conditionals, the sensitivity and detection limit of the colorimetric assay were demonstrated. Figure 3 shows the color and absorption change of AuNPs with the addition of the mixture of peptide and different amounts of DPP-IV. The colorimetric assay allowed for the determination of DPP-IV concentration as low as 5 mU/mL for rapid and reliable visualization by comparison with the blank sample (Figure 3A). Furthermore, the concomitant absorbance change was monitored by UV/Vis spectroscopy. As shown in Figure 3B, increase of DPP-IV concentration induced the decrease in the absorbance at 630 nm. The dependence of the A_630_/A_520_ on the DPP-IV concentration was shown in Figure 3C. It can be observed that A_630_/A_520_ decreased linearly with DPP-IV concentration in the range of 0.1–50 mU/mL. The linear regression equation can be expressed as A_630_/A_520_ = 0.725 [DPP-IV] (mU/mL) − 0.008, R = 0.993. The detection limit was determined to be 70 μU/mL based on the slope of the dose-response curve and the standard deviation of blank responses (*n* = 11). It has been suggested that DPP-IV inhibitor can increase the half-life of incretins, decrease the concentration of plasma glucose, and improve impaired glucose tolerance [2]. To demonstrate that the colorimetric method could be used for screening of DPP-IV inhibitor, diprotin A (a well-known DPP-IV inhibitor) was tested. As shown in Figure 3D, the inhibitor ratio increased with the increasing concentration of inhibitor in the range of 100~1000 μM. The maximum inhibition of diprotin A was found to be 92.1%. The half-maximal inhibitory concentration (IC_50_) for 50 mU/mL DPP-IV was determined to be approximately 577 μM.

### 2.3. Electrochemical Assay of DPP-IV

The principle of the electrochemical strategy for assay of DPP-IV activity is presented in Scheme 2. The SAM of peptide/6-mercapto-1-hexanol (MCH) formed on the gold electrode surface behaves as a barrier for [Fe(CN)_6_]^3−^/[Fe(CN)_6_]^4−^. However, the sensing electrode can capture AuNPs and free tripeptide in the solution through the peptide-AuNPs-tripeptide interaction. Then, surface-tethered peptide-AgNPs-tripeptide recruited more AuNPs as well as tripeptide, resulting in the formation of a network of AuNPs-tripeptide on the electrode surface. The unique electrical properties of AuNPs may lead to a significant fall of charge transfer resistance [41,42,43]. Once the peptide ether on the electrode surface or in the solution was clipped by DPP-IV, it would lose the ability to trigger the assembly of AuNPs on the electrode surface. Thus, the liquid-phase colorimetric assay was converted into an electrochemical assay.

**Figure 4:**
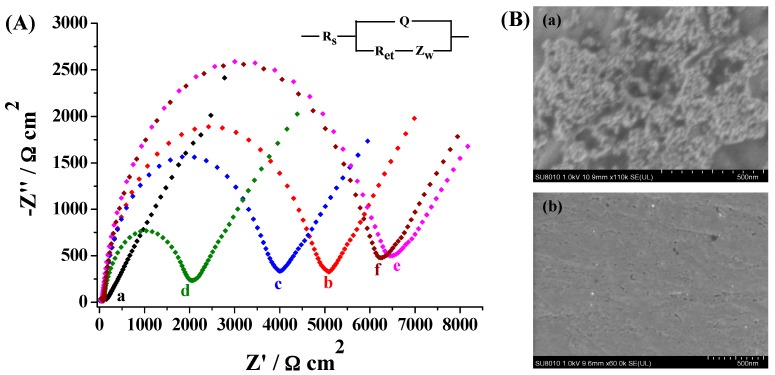
(**A**) Electrochemical impedance spectroscopy (EIS) of the bare electrode (curve a) and peptide/6-mercapto-1-hexanol (MCH)-modified electrode before (curve b) and after incubation with AuNPs (curve c) or AuNPs/peptide (curve d). Curve e corresponds to that after incubation with DPP-IV only. Curve f corresponds to that of incubation with AuNPs/peptide following the incubation with DPP-IV. The final concentrations of AuNPs, peptide and DPP-IV were 1.8 nM, 0.4 μM and 5 mU/mL, respectively; (**B**) SEM images of peptide/MCH-modified electrode incubated with AuNPs/peptide before (panel a) and after treatment (curve b) by DPP-IV.

Electrochemical impedance spectroscopy (EIS) was used herein to monitor the change of surface properties. The impedance spectra were fitted with a Randles equivalent circuit, including the electrolyte resistance between working and reference electrodes (R_s_), the Warburg impedance (Z_w_), a constant phase element representing the double layer capacitance for an unmodified electrode or the capacitance of the self-assembled monolayers for the modified electrodes (Q) and the electron-transfer resistance (R_et_) (see the inset in Figure 4A). The R_et_ on the peptide/MCH-covered electrode (curve b) was significantly higher than that on the bare gold electrode (curve a). The result is understandable since the peptide/MCH SAM can repulse [Fe(CN)_6_]^3−^/[Fe(CN)_6_]^4−^ from the electrode surface [44]. However, after incubating the peptide/MCH-covered electrode with AuNPs/peptide (curve d), the R_et_ decreased significantly. Note that there is no apparent resistance change when incubating the electrode with peptide only (data not shown) and a smaller decrease in R_et_ was observed when incubating the electrode with AuNPs alone (curve c). Thus, the significant decrease in the R_et_ of curve d was attributed the formation of a network of AuNPs. Furthermore, there is an increase in R_et_ when incubating the sensing electrode with DPP-IV (curve d), demonstrating that remove of one positively charged arginine residue from the electrode surface caused the change of surface properties. However, no significant decrease in R_et_ was observed when incubating the DPP-IV-treated electrode with AuNPs/peptide (curve f), and the R_et_ in this case was greatly higher than that of curve d. Thus, cleavage of the peptide by DPP-IV made the assembly of AuNPs on electrode surface impossible. This was further confirmed by the SEM observation. As shown in Figure 4B, a network of AuNPs was formed on the peptide/MCH-covered sensing electrode surface (panel a), but fewer AuNPs were absorbed on the DPP-IV-treated sensing electrode surface (panel b). These results were acceptable since the cleavage products showed no or poor binding to AuNPs and could not crosslink AuNPs, which could be demonstrated by the above colorimetric assay. These results confirmed that the liquid-phase colorimetric assay was developed into an electrochemical analysis.

### 2.4. Optimization of Experimental Conditions for Impedance Assay

**Figure 5:**
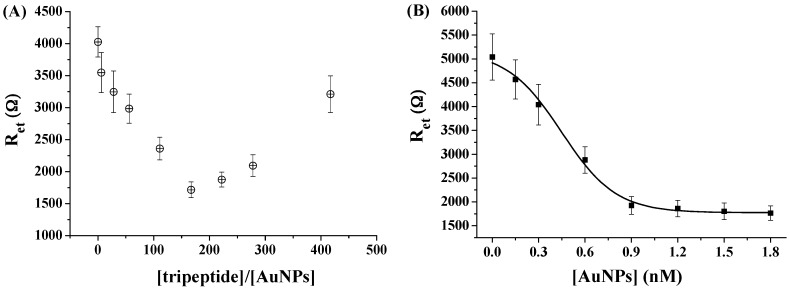
(**A**) Dependence of R_et_ on the concentration ratio of the tripeptide to AuNPs. The final concentration of AuNPs was 1.8 nM and that of the peptide varied from 0 to 0.75 μM (0, 0.01, 0.05, 0.1, 0.2, 0.3, 0.4, 0.5 and 0.75 μM); (**B**) Dependence of R_et_ on the AuNPs concentration. The concentration ratio of the tripeptide to AuNPs kept at 167.

Higher concentration of tripeptide (Arg-Pro-Arg) can make the AuNPs aggregation more powerful. However, higher concentration of tripeptide in the solution would compete with the immobilized peptide substrate to bind AuNPs, thus deteriorating the assembly of AuNPs. To facilitate the electrochemical detection of DPP-IV, we first investigated the influence of the concentration ratio of peptide to AuNPs (tripeptide)/(AuNPs) on the R_et_ value. It was found that R_et_ was initially decreased with the increasing (tripeptide)/(AuNPs) ratio until the minimum value appeared at 167, followed by a sharp increase (Figure 5A). Also, the influence of the AuNPs/tripeptide concentration on the electrochemical impedance was investigated. As a result, R_et_ decreased with the increase of AuNPs/tripeptide concentration and began to level off beyond 0.9 nM (AuNPs concentration) (Figure 5B). Therefore, in the following detection assay, the concentrations of the AuNPs and tripeptide were kept at 1.2 nM and 0.2 μM, respectively.

### 2.5. Sensitivity to DPP-IV

**Figure 6:**
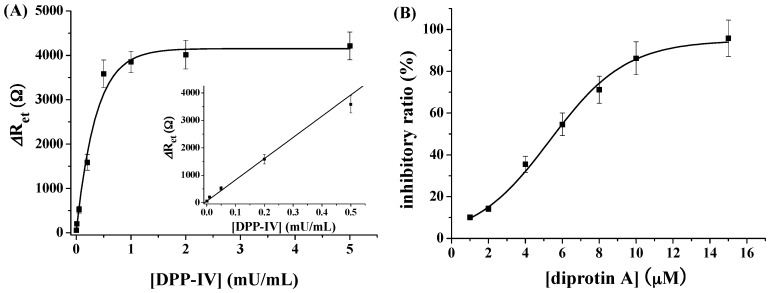
(**A**) Dependence of ΔR_et_ on DPP-IV concentration (0, 0.001, 0.01, 0.05, 0.2, 0.5, 1, 2 and 5 mU/mL); (**B**) Inhibitory ratio of different concentrations of diprotin A (1, 2, 4, 6, 8, 10 and 15 μM) on the DPP-IV (0.5 mU/mL) activity.

Under the optimized experimental conditions, DPP-IV at various concentrations was analyzed. The quantitative assay was conducted by measuring the change of electron transfer resistance (ΔR_et_ = R′_et_ − R_et_, where R′_et_ and R_et_ represent the electron transfer resistance of the sensing electrode with and without treatment by DPP-IV, respectively, followed by incubation with AuNPs/peptide). As shown in Figure 6A, ΔR_et_ increased with increasing DPP-IV concentration (DPP-IV). It was proportional to (DPP-IV) in the range from 0.001 to 0.5 mU/mL. The regression equation was ΔR_et_ = 7738 (DPP-IV) (mU/mL) + 56, R = 0.977. The detection limit of this electrochemical method was calculated to be 0.55 μU/mL. The value was approximately 2 orders of magnitude lower than that obtained by the aforementioned AuNP-based colorimetric method (70 μU/mL), demonstrating that AuNP-based liquid-phase colorimetric assay has been converted into the electrochemical assay with improving sensitivity. The detection limit of our electrochemical method is comparable to that (39 nU/mL) achieved on the AuNPs-modified electrode by using a ferrocence-labeled peptide as the probe [12]. Furthermore, the electrochemical method was used to determine DPP-IV inhibitor. It was found that the R_et_ decreased with the increasing concentration of diprotin A in the range of 1–15 μM. The maximum inhibition was found to be 95.8% with the IC_50_ value of ~5.6 μM (Figure 6B).

## 3. Materials and Methods

### 3.1. Reagents and Materials

Peptides with the sequences of Pro-Arg, Arg-Pro-Arg and Arg-Pro-Arg-Pro-Pro-Pro-Pro-Cys (≥98%) were synthesized and purified by Synpeptide Co., Ltd. (Shanghai, China). DPP-IV, diprotin A, tris(2-carboxyethyl)phosphine hydrochloride (TCEP), trisodium citrate, 6-mercapto-1-hexanol (MCH), KH_2_PO_4_ and K_2_HPO_4_ were purchased from Sigma-Aldrich (Shanghai, China). Other reagents were analytical-grade and used without additional treatment. The peptide stock solution at the concentration of 2 mM was dissolved with deionized water and diluted with a phosphate-buffered saline solution (PBS buffer, 2 mM, pH 7.6) before use. The citrate-stabilized AuNPs with a size of 13 nm were prepared using a trisodium citrate reduction method [45]. The concentration of synthesized AuNPs was determined by Beer’s law with an extinction coefficient of 2.7 × 10^8^ M^−1^ cm^–1^ [18]. Unless otherwise noted, the reactions were conducted at room temperature.

### 3.2. Instruments

The UV/Vis spectra were collected on a Cary 60 spectrophotometer using a 1 cm quartz spectrophotometer cell. The photograph was taken by a digital camera. The transmission electron microscope (TEM) images were taken by an FEI Tecnai G2 T20 TEM (Hillsboro, OR, USA). Scanning electron microscope (SEM) images were taken on a Hitachi SU8010 SEM (Tokyo, Japan). Dynamic light scattering (DLS) and zeta potentials (ζ) were measured on a Malvern Zetasizer Nano-ZS (Malvern, Worcetershire, UK). The enzymatic products were characterized by mass spectroscopy (LCT Premier XE, Waters, Milford, MA, USA). The electrochemical experiments were carried out on a CHI 660E (CH Instruments, Inc., Shanghai, China) electrochemical workstation. The auxiliary electrode is a platinum wire. The reference electrode is Ag/AgCl.

### 3.3. Colorimetric Assay of DPP-IV Activity

To investigate the effect of peptide Arg-Pro-Arg on the AuNPs aggregation, 250 μL of AuNPs suspension was added to 250 μL of a known concentration of peptide solution. After incubation for 5 min, color change was observed with the naked eyes and the photograph was taken by a digital camera. UV/Vis absorption spectra were collected with the spectrophotometer. To determine the activity of DPP-IV, 200 μL of peptide solution was incubated with 50 μL of DPP-IV solution in a test tube at room temperature. After reaction for a given time, the solution was mixed with 250 μL of AuNPs for 5 min. The color and absorption change of the mixture were then measured by the digital camera and the UV/Vis spectrophotometer, respectively.

To evaluate the inhibition ability of the potential inhibitor, DPP-IV was first mixed with the tested inhibitor for 30 min. Then, peptide was added into the mixture to react for 1 h. The other detection procedures are the same as those for the detection of DPP-IV. The inhibitory ratio (%) of the potential inhibitors on enzymatic activity was expressed as follows: inhibitory ratio (%) = (R_3_ − R_2_)/(R_1_ − R_2_) × 100%, where R_1_ is the A_630_/A_520_ ratio of AuNPs/peptide in the absence of DPP-IV, R_2_ is the A_630_/A_520_ ratio of AuNPs/peptide in the presence of DPP-IV, R_3_ is the A_630_/A_520_ ratio of AuNPs/peptide in the presence of DPP-IV and inhibitor.

### 3.4. Electrochemical Assay of DPP-IV Activity

The cleaned polycrystalline gold disk electrode was placed in a 100 μL PBS solution comprising of 10 μM cysteine-containing peptide (Arg-Pro-Arg-Pro-Pro-Pro-Pro-Cys) and 50 mM TCEP for 12 h. The peptide was assembled onto the electrode surface through the Au-S interaction. Insertion of the four proline residues between the substrate and the cysteine residue could help position the peptide away from the electrode surface to achieve a higher enzyme cleavage efficiency [46,47]. After the formation of the peptide self-assembled monolayers (SAMs) on electrode surface, the electrode was soaked in a solution of 1 mM MCH for 30 min. Then, the electrode was rinsed thoroughly with ethanol and water to remove non-specifically adsorbed substance. Modification of electrode with MCH can block the unreacted gold surface and displace non-specifically adsorbed peptide. For the assay of DPP-IV activity, the peptide/MCH-covered electrode was first pre-incubated with 50 μL of DPP-IV solution in a homemade plastic cell for 30 min. Then, 50 μL of peptide (Arg-Pro-Arg) solution was added into the tube for 60 min-incubation, followed by addition of 100 μL of AuNPs suspension. After incubation for 10 min again, the electrode was rinsed thoroughly with water and then placed in the electrolyte for impedance measurement in the frequency range of 0.01–500 kHz. The electrolyte was comprised of 10 mM [Fe(CN)_6_]^3−^/[Fe(CN)_6_]^4−^ (1:1) and 0.5 M KCl and the potential was set at 0.245 V.

To evaluate the inhibition ability of the potential inhibitors, DPP-IV was first mixed with the tested inhibitor for 10 min. The other detection procedures were the same as those for the detection of DPP-IV. The inhibitory ratio (%) of the potential inhibitors on enzymatic activity was expressed as follows: inhibitory ratio (%) = (R_et_^3^ − R_et_^2^)/(R_et_^1^ − R_et_^2^) × 100%, where R_et_^1^ is the electron-transfer resistance in the case without DPP-IV, R_et_^2^ is the electron-transfer resistance in the case with DPP-IV, R_et_^3^ is the electron-transfer resistance in the case with DPP-IV and inhibitor.

## 4. Conclusions

In summary, we presented the colorimetric and electrochemical methods for DPP-IV activity assay and inhibitor screening based on the unique size-dependent optical property and the good electrical ability of AuNPs. DPP-IV could be detected with a detection limit of 70 μU/mL by UV/vis spectra or 0.55 μU/mL by the electrochemical method. The proposed methods could also be used for determining DPP-IV inhibitor. Compared with the traditional methods, the colorimetric method requires very simple detection procedure and minimum instrumental investment. Furthermore, the AuNP-based liquid-phase colorimetric method could be developed into an enhanced surface tethered electrochemical method. Such conversion improved the sensitivity and maintained the simple detection principle and easy manipulation procedure of the colorimetric method. We believe that this work should be valuable for detection of DPP-IV activity, screening of novel DPP-IV inhibitors and design of new peptidases biosensors.
